# SETDB1 Regulates Porcine Spermatogonial Adhesion and Proliferation through Modulating *MMP3/10* Transcription

**DOI:** 10.3390/cells11030370

**Published:** 2022-01-22

**Authors:** Ruifang Liu, Zidong Liu, Ming Guo, Wenxian Zeng, Yi Zheng

**Affiliations:** Key Laboratory for Animal Genetics, Breeding and Reproduction of Shaanxi Province, College of Animal Science and Technology, Northwest A&F University, Yangling 712100, China; liuruifang79@163.com (R.L.); liuzidong96@foxmail.com (Z.L.); guoming9979@126.com (M.G.)

**Keywords:** SETDB1, MMP10, pig, spermatogonial stem cell (SSC), homing process, cell adhesion

## Abstract

The transition from gonocytes into spermatogonia takes place during the homing process. A subpopulation of undifferentiated spermatogonia in niche then shifts to spermatogonial stem cells (SSCs), accompanied by the self-renewal ability to maintain life-long fertility in males. Enormous changes in cell morphology, gene expression, and epigenetic features have been reported during spermatogenesis. However, little is known about the difference of these features in SSCs during aging. Here, we examined the dynamics of SET domain bifurcated 1 (SETDB1) expression in porcine testes. SETDB1 was expressed in postnatal undifferentiated spermatogonia, while gradually disappeared after being packed within the basal compartment of seminiferous tubules. In addition, the cell-adhesion ability, proliferative activity, and trimethylation of the histone H3 lysine 9 (H3K9me3) level were significantly altered in SETDB1-deficient porcine SSCs. Moreover, the matrix metalloproteinases 3/10 (MMP3/10) was upregulated at both mRNA and protein levels. These results illustrate the significance of SETDB1 in modulating early male germ cell development.

## 1. Introduction

The proliferation and differentiation of spermatogonial stem cells (SSCs) are the basis of mammalian spermatogenesis. The isolation and long-term propagation of rodent SSCs have been well studied. After 2 years of propagation in vitro, the transplanted murine SSCs are still capable of forming colonies at the basement membrane of seminiferous tubules and of further differentiating into fertile sperm [1]. In additional to the optimized in vitro culture condition, the identity and molecular characterization of SSCs have been revealed in recent studies using knockout mouse models [2]. In testes, Sertoli cells, Leydig cells and peritubular myoid cells play important roles in the maintenance of SSCs [3,4,5,6]. Sertoli cells support germ cells and regulate the proliferation of SSCs by paracrine glial cell line-derived neurotrophic factor (GDNF), basic fibroblast growth factor (bFGF) and WNT [7,8]. Peritubular myoid cells influence the proliferation of germ cells by secreting GDNF, bFGF, Insulin-like growth factor 1 (IGF1) and various extracellular matrix such as laminin, fibrin and collagen [3,6,9]. The production of colony stimulating factor 1 (CSF1) and testosterone by Leydig cells stimulates SSCs and Sertoli cells to directly and indirectly regulate the proliferation of SSCs [4,10]. In addition, adhesion molecules, such as β1-integrin, ensure the proper localization of cells in seminiferous tubules and the homing of SSCs [11], suggesting the significance of cell–cell interaction and adhesion in spermatogenesis. The GDNF receptor signaling pathway is a key pathway for SSC self-renewal and proliferation [7,12,13,14]. Recent studies have shown that GDNF does not affect the self-renewal of porcine SSCs, and that the expression of GDNF receptor GFRα1 in porcine testes is also significantly lower than that in mice [15]. SSCs from large domestic animals hold some unique characteristics. However, research on homing and proliferation of porcine SSCs is scarce.

Epigenetic regulation plays an important role in spermatogenesis and in male reproduction. The post translational modifications (PTMs) on histone, including methylation, acetylation, phosphorylation, ubiquitination, SUMOylation, glycosylation, and ADP-ribosylation, regulate the transcription activity of genes involved in mitosis and meiosis [16,17,18]. The chromatin accessibility shows little difference between human stage-specific embryonic antigen-4 positive (SSEA4^+^) undifferentiated spermatogonia and cluster of differentiation 117 positive (c-KIT^+^) differentiating spermatogonia, as revealed by assay for transposase-accessible chromatin using sequencing (ATAC-seq) [19]. By contrast, the chromatin accessibility changes dramatically during murine male germline development from spermatogonia to spermatocyte [20]. The methylation on histone H3 lysine 4 (H3K4) that is associated with transcriptional activation modulates spermatogenesis and germ cell maintenance [21]. Deficiency in H3K4 demethylase KDM1A activates SSC differentiation, meiotic initiation and misexpression of differentiation genes [22], while methyltransferase KMT2B depletion perturbs differentiation of spermatogonia [21].

SET domain bifurcated 1 (SETDB1), also termed ESET and KMT1E, is an important H3K9me3-catalyzing methyltransferase responsible for endogenous retrovirus (ERV) silencing in somatic [23] and germ cells [24,25]. Ectopic SETDB1 expression in colorectal cancer activates the STAT1-CCND1/CDK6 axis, resulting in accelerated proliferation by impeding the cell cycle transition from G0/G1 phase to S phase [26]. Overexpression of SETDB1 also promotes G1/S phase transition in nasopharyngeal carcinoma [27], while suppression of SETDB1 reduces the cell migratory and clonogenic ability in glioma cells [28].

Consistently, SETDB1 is crucial to germline development and functions differentially at different stages. Knockdown (KD) of SETDB1 in primordial germ cells (PGCs) derepresses the H3K9me3-modified ERVs, resulting in reduced DNA methylation and postnatal hypogonadism [24]. SETDB1 maintains the survival of murine SSCs by regulating the H3K9me3 level in promoter regions of genes downstream from the PTEN/AKT/FOXO1 pathway [29,30]. Furthermore, meiotic sex chromosome inactivation (MSCI) in mid-pachytene spermatocytes is dependent on the H3K9me3 deposition specifically by SETDB1 [25]. While it is a common phenotype that the cell proliferation is positively associated with SETDB1, how SETDB1 regulates SSC propagation is not known.

Here, we showed that the expression pattern of SETDB1 dynamically changed in postnatal, pubertal, and adult porcine testes. We then investigated the role of SETDB1 in modulating the porcine SSC proliferative activity and cell-adhesion ability, with an aim to better understand the epigenetic regulation in germ cell development.

## 2. Materials and Methods

### 2.1. Experimental Design

Experiment 1 was designed to investigate the expression pattern of SETDB1 in postnatal, pubertal, and adult porcine spermatogonia and spermatocytes. Immunofluorescence analysis was performed on porcine testis sections.

Experiment 2 was devised to detect the phenotypes of SETDB1-KD porcine SSCs. The immortalized porcine SSC line was transfected with small interfering RNAs (siRNAs) to knock down the expression of SETDB1. The knockdown efficiency was detected by quantitative reverse transcription polymerase chain reaction (qRT-PCR) and Western blot. The phenotypes of SETDB1-KD porcine SSCs were identified by EdU staining assay, cell cycle analysis and cell-adhesion assay.

Experiment 3 sought to elucidate the role of SETDB1 in modulating the cell-adhesion ability of porcine SSCs. The expression of targeted genes was detected by qRT-PCR and Western blot analysis. The H3K9me3 level in SETDB1-KD SSCs was detected by Western blot analysis.

### 2.2. Animals

Guanzhong black pigs, 7 d (postnatal, BW = 2.8 ± 0.3 kg, *n* = 3), 60 d (pubertal, BW = 20 ± 1 kg, *n* = 3) and 150 d (adult, BW = 50 ± 4 kg, *n* = 3), were castrated on a local farm (Besun farm, 107° east longitude and 34° north latitude, Yangling, China) in June or August, and the testicle samples were collected by farm personnel on researchers’ request. The testis samples were washed with 75% ethanol and phosphate-buffered saline (PBS). The testis samples were then dissected, fixed in 4% paraformaldehyde (PFA) for 10–14 h at room temperature, dehydrated in 30%, 50%, 70%, 80%, 90% and 100% ethanol successively (30 min for each), and embedded in paraffin.

### 2.3. Immunofluorescence Analysis

For immunofluorescence (IF) analysis, 5 μm testicular sections were prepared, followed by deparaffination and rehydration. For immunocytofluorescence (ICF), 3 × 10^4^ cells were fixed with 4% PFA and pipetted onto pre-coated poly-lysine (Sigma, St. Louis, MO, USA) glass slides at 100 μL. Then, slides were permeabilized with 0.1% TritonX-100 (MP Biomedicals, Illkirch, France), and blocked with SuperBlock Blocking Buffer (Thermo Scientific, Rockford, IL, USA) for 2 h at room temperature. Testicular sections/cells were then incubated with primary antibodies diluted in buffer at 4 °C overnight. Specifically, the primary antibodies used were rabbit anti-SETDB1 (1:100; 93212, Cell Signaling Technology, Danvers, MA, USA), mouse anti-SETDB1 (1:30; sc-166621, Santa Cruz Biotechnology, Dallas, TX, USA), rabbit anti-H3K9me3 (1:50; 13969, Cell Signaling Technology), mouse anti-UCH-L1 (1:200; ab8189, Abcam, Cambridge, UK), goat anti-SYCP3 (1:20; AF3750, R&D system, NE Minneapolis, MN, USA), rabbit anti-VASA (1:200; ab13840, Abcam), rabbit anti-PLZF (1:200; ab104854, Abcam), rabbit anti-SV40 (1:200; 15729, Cell Signaling Technology), mouse anti-PCNA (1:200; sc-56, Santa Cruz Biotechnology), and mouse anti-IgG (1:200; sc-2025, Santa Cruz Biotechnology). Next day, after repeated wash with DPBS, testicular sections/cells were incubated with Alexa fluor 488/594 conjugated secondary antibody (Yeasen, Shanghai, China) for 2 h at 4 °C, followed by nuclear staining with DAPI (Bioworld Technology, St. Louis Park, MN, USA). Images were captured with a fluorescence microscope camera (Nikon Eclipse 80i, Tokyo, Japan). For each testis sample, 30 round and intact cross-sections of seminiferous tubules were analyzed.

### 2.4. Cell Culture

The cell line used in this study was the immortalized porcine SSC line established by us [31]. The cell line was characterized by immunofluorescence staining of different SSC markers. The cells were cultured in DMEM/high glucose (Gibco, Grand Island, NY, USA) containing 5% fetal bovine serum (Gibco, Mesenchymal Stem Cell FBS Qualified), 5% knockout serum replacement (KSR; Gibco), 100 unit/mL penicillin and streptomycin (Hyclone, Logan, Utah), 1 × MEM vitamin solution (Gibco), 1× non-essential amino acid (NEAA; Gibco), 2 mmol/L Glutamax (Gibco), 40 ng/mL recombinant human GFRA1 (BioLegend, San Diego, CA, USA), 20 ng/mL recombinant human GDNF (Peprotech, Rocky Hill, NJ, USA) and 10 ng/mL recombinant human bFGF (Peprotech). The cells were maintained at 37 ℃ in a humidified incubator containing 5% CO_2_.

### 2.5. SETDB1-siRNA Transfection

*SETDB1* siRNAs, i.e., *SETDB1*-1 and *SETDB1*-2, were synthesized by GenePharma (Shanghai, China). Sequences of the porcine *SETDB1* siRNAs are shown in Table S1. The Advanced Transfection Reagent (Zeta Life, Menlo Park, CA, USA) was used for siRNA transfection. The cells were transfected with SETDB1 siRNAs for 48 h or 72 h, and were then collected for qRT-PCR and Western blot analyses.

### 2.6. qRT-PCR Analysis

The RNAs of samples were extracted using TRIzol reagent (Invitrogen, Vilnius, Lithuania). The Revert Aid First Strand cDNA Synthesis Kit (Roche, Mannheim, Germany) was used for reverse transcription. All specific primers for each gene were designed using the Primer Premier 5 software (Premier Biosoft International, Palo Alto, CA, USA) and are shown in Table S1. Then, FastStart Universal SYBR Green Master (Roche) was used for real-time quantitation of mRNA levels, using an iQ5 detection system (Bio-Rad, Hercules, CA, USA). All data were calculated by using the comparative Ct-method. All expression value was normalized to hypoxanthine phosphoribosyl transferase 1 (HPRT-1). For significance analysis, the qRT-PCR data are processed at a log2 scale.

### 2.7. Western Blot Analysis

A RIPA Kit (Beyotime, Nanjing, China) was used to collect the protein lysates. Collected protein lysates were separated by polyacrylamide gel electrophoresis in the presence of sodium dodecyl sulfate (SDS-PAGE) and transferred to poly-vinylidene fluoride (PVDF) membranes. Membranes were blocked with 5% non-fat milk powder dissolved in Tris-buffered saline containing 0.1% tween-20 (TBST) at room temperature for 2 h. Blots were incubated with primary antibodies diluted in primary antibody dilution buffer (Coolaber, Beijing, China) at 4 °C overnight. Specifically, rabbit anti-SETDB1 (1:1000; 93,212, Cell Signaling Technology), rabbit anti-H3K9me3 (1:1000; 13969, Cell Signaling Technology), mouse anti-ACTB (1:3000; CW0096, CWBIO), mouse anti-MAD2 (1:500; sc-374131, Santa Cruz Biotechnology), mouse anti-MMP3/10 (1:1000; sc-374029, Santa Cruz Biotechnology) and rabbit anti-Histone H3 (1:1000; 17168-1-AP, Proteintech Group) were employed as the primary antibodies. After washing three times with TBST, membranes were incubated with HRP conjugated secondary antibody (Yeasen) for 2 h at room temperature. Membranes were again washed with TBST for three times. Protein expression was detected using an ECL imaging system (Millipore, Burlington, MA, USA) and viewed using a ChemiDox XRS (Bio-Rad) system. To examine the expression of protein in different cell fractions, we normalized the target band density with that of ACTB or histone H3.

### 2.8. EdU Staining Assay

Cells were seeded into 96-well plates and transfected with siRNAs. EdU staining was performed using the Cell-Light EdU Apollo488 in vitro Kit (RiboBio, Guangzhou, China), according to the manufacturer’s protocol. All images were acquired using a fluorescence microscope (Tokyo, Japan).

### 2.9. Cell Cycle Analysis

Cells were suspended in 75% cold ethanol for 1 h and permeabilized with 0.1% Triton X-100 and RNase (100 μg/mL) for 30 min. Then, the cells were stained with 10 μg/mL DAPI for 30 min. The DNA content was detected by flow cytometry (BD FACS Aria III, United States), and the corresponding data were analyzed with ModFit LT 5.0 (Verity Software House, Topsham, ME, USA).

### 2.10. Cell-Adhesion Assay

The 96-well plates were first coated with 10 μg/mL fibronectin. Then, cells were transfected with siRNAs for 60 h and seeded into pre-coated 96-well plates at 37 °C for 2 h. After 30 min incubation, we removed the culture medium and washed the non-adherent cells with DPBS. Residual cells were then counted using CCK-8 assay (Beyotime).

### 2.11. StatisticalAanalysis

All data were collected from at least three independent experiments. Data were analyzed using unpaired *t*-test or one-way ANOVA analysis followed by Bonferroni multiple-comparison test (SPSS 26 for windows, IBM, Armonk, NY, USA). Significance was presented as * *p* < 0.05, ** *p* < 0.01 or *** *p* < 0.001. Error bars represented the standard error of mean (SEM).

## 3. Results

### 3.1. Characterization of SETDB1 during Porcine Spermatogenesis

**Figure 1:**
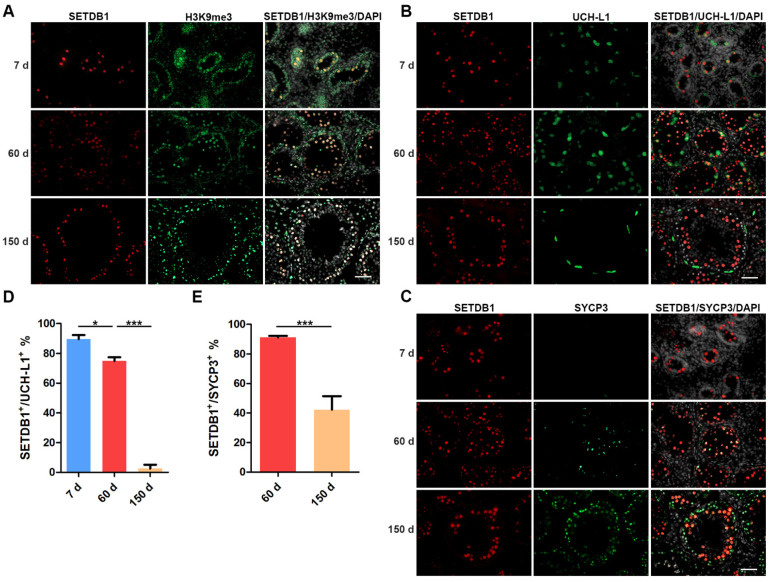
SETDB1 expression in porcine testes. (**A**–**C**) Immunofluorescence panel of SETDB1 (red), H3K9me3 (green), UCH-L1 (green), SYCP3 (green), and DAPI (white) at 7, 60 and 150 days. Scale bar = 100 μm. (**D**) The proportion of SETDB1^+^ cells in undifferentiated spermatogonia (UCH-L1^+^). (**E**) The proportion of SETDB1^+^ cells in spermatocytes (SYCP3^+^). Significance was presented as * *p* < 0.05 or *** *p* < 0.001. Error bars represented the standard error of the mean (SEM).

We first detected the localization of SETDB1 and H3K9me3 in 7 d, 60 d and 150 d Guanzhong black pig testes. We found that SETDB1^+^ cells stained heavily for H3K9me3 (Figure 1A), in line with the methyltransferase function of SETDB1. The expression of SETDB1 has been reported to differ among spermatogenic subtypes [32]. Ubiquitin carboxy-terminal hydrolase-L1 (UCH-L1) was expressed in gonocytes and undifferentiated spermatogonia including SSCs [33,34,35], while synaptonemal complex protein 3 (SYCP3) is specifically expressed in spermatocytes [25,36]. These marker proteins were used to identify gonocytes, spermatogonia and spermatocytes.

In 7 d porcine testes that only contain gonocytes and no SYCP3^+^ cells were found (Figure 1B,C). The majority of gonocytes were positive for UCH-L1 and SETDB1, and SETDB1^+^ UCH-L1^−^ cells were also found (Figure 1B,D). The proportion of SETDB1^+^/ UCH-L1^+^ cells was significantly reduced at 60 d, while 91.09% of SYCP3^+^ spermatocytes expressed SETDB1 (Figure 1C,E). Notably, UCH-L1^+^ gonocytes and undifferentiated spermatogonia showed visibly weaker SETDB1 staining than other SETDB1^+^ cells, and stronger SETDB1 staining could be detected in SYCP3^+^ spermatocytes. The elongated spermatids appeared in 150 d testes, although SETDB1 staining was preferentially present in the SYCP3^+^ spermatocytes, and the proportion of SETDB1^+^/ SYCP3^+^ cells was significantly decreased compared with that at 60 d. Interestingly, SETDB1 and UCH-L1 staining barely overlapped at this stage, with only few undifferentiated spermatogonia weakly expressing both SETDB1 and UCHL-1 (Figure 1B). The discrepancy of the SETDB1 expression in 7 d, 60 d, and 150 d UCH-L1^+^ germ cells indicated that SETDB1 might regulate specific biological processes before the initiation of spermatogonial differentiation.

### 3.2. Knockdown (KD) of SETDB1 via siRNAs in Porcine SSCs

**Figure 2:**
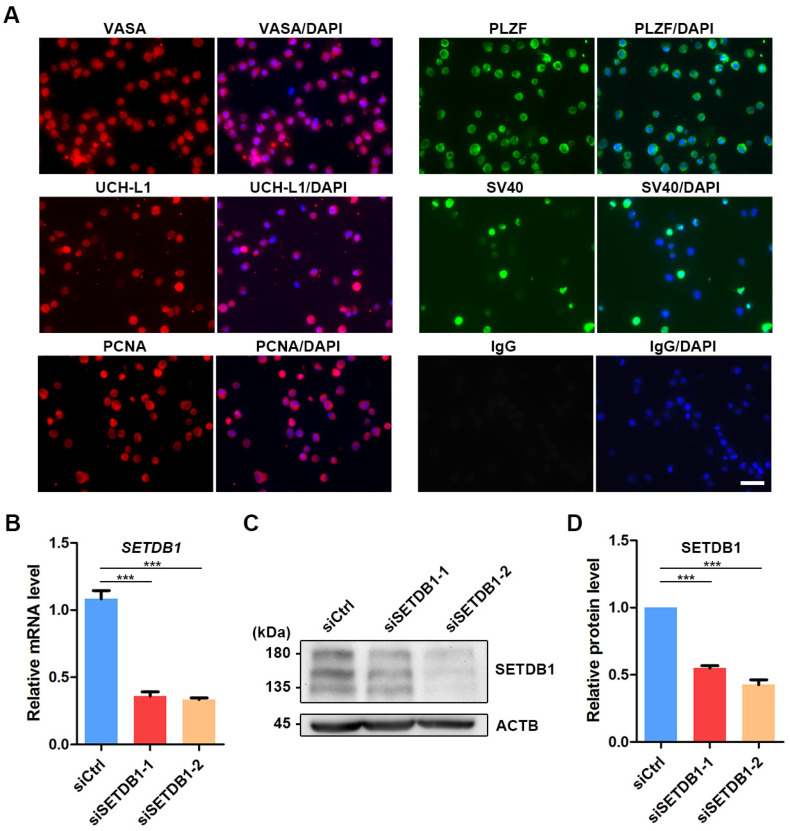
Knockdown of SETDB1 in porcine SSCs. (**A**) Immunofluorescence panel of VASA (red), PLZF (green), UCH-L1 (red), SV40 (green), PCNA (red), negative control (IgG; green) and DAPI (blue) in porcine SSCs. Scale bar = 50 μm. (**B**) qRT-PCR analysis of the relative mRNA level of *SETDB1* in porcine SSCs. (**C**) Western blot analysis of the protein levels of SETDB1 and ACTB in porcine SSCs. (**D**) The protein level of SETDB1 relative to ACTB. Significance was presented as *** *p* < 0.001. Error bars represented the standard error of the mean (SEM).

To investigate the function of SETDB1 in porcine SSCs, we knocked down SETDB1 in porcine SSCs by siRNAs [31,37]. The immortalized porcine SSC line was established by stably expressing the simian virus 40 (SV40) large T antigen (Figure 2A) [31]. The pan-germ cell marker VASA and SSC markers promyelocytic leukemia zinc finger (PLZF) and UCH-L1 were found expressed in porcine SSCs (Figure 2A). The expression of proliferating cell nuclear antigen (PCNA) indicated that the proliferative property was preserved in the immortalized porcine SSC line (Figure 2A). The knockdown efficiency of SETDB1 was then detected by qRT-PCR and Western blot. The expression of SETDB1 was significantly reduced at both mRNA (Figure 2B) and protein levels (Figure 2C,D).

### 3.3. SETDB1-KD Impaired Cell Proliferation and Cell Cycle Transition

**Figure 3:**
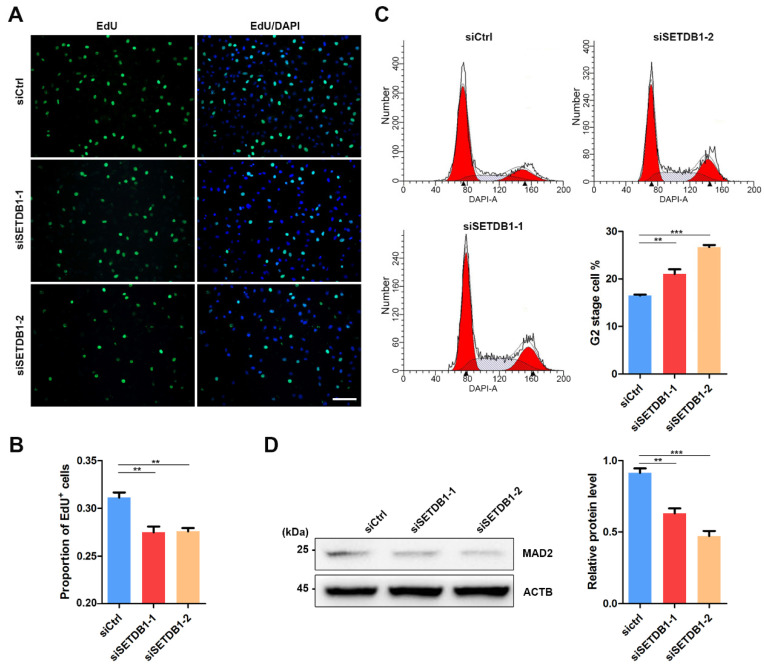
Knockdown of SETDB1 impaired cell proliferation and cell cycle transition. (**A**) Representative images of EdU incorporation in siSETDB1 and siCtrl cells. The proliferative cells were detected by Cell-Light EdU Apollo488 (green), and the nucleus was labelled with DAPI (blue). (**B**) Quantification of EdU incorporation in siSETDB1 and siCtrl cells. (**C**) Cell cycle analysis of siSETDB1 and siCtrl cells using flow cytometry. Proportion of the G2 stage cells is shown in the column diagram. The blank triangle indicates the peak of the Y-axis. (**D**) Western blot analysis of the protein levels of MAD2 and ACTB in porcine SSCs. The protein level of SETDB1 relative to ACTB is shown in the column diagram. Significance was presented as ** *p* < 0.01 or *** *p* < 0.001. Error bars represented the standard error of the mean (SEM).

The expression of SETDB1 is highly correlated with cell proliferation in cancer cells [27,38,39,40]. Consistently, we found that the morphology of SETDB1-KD cells was visibly different from that in the siCtrl group. EdU incorporation assay showed that the cell proliferation was significantly decreased in SETDB1-KD cells (Figure 3A,B). Flow cytometry analysis demonstrated that SETDB1-KD led to cell arrest at the G2 stage (Figure 3C). Mitotic arrest deficient 2 (MAD2) is a core regulator of spindle assembly checkpoint-related proteins involved in G2/M transition [41,42]. Consistently, the expression of MAD2 was significantly reduced in SETDB1-KD cells (Figure 3D), indicating that SETDB1 affects G2/M transition in porcine SSCs.

### 3.4. SETDB1-KD Affected Cell Adhesion and Cell Spread

**Figure 4:**
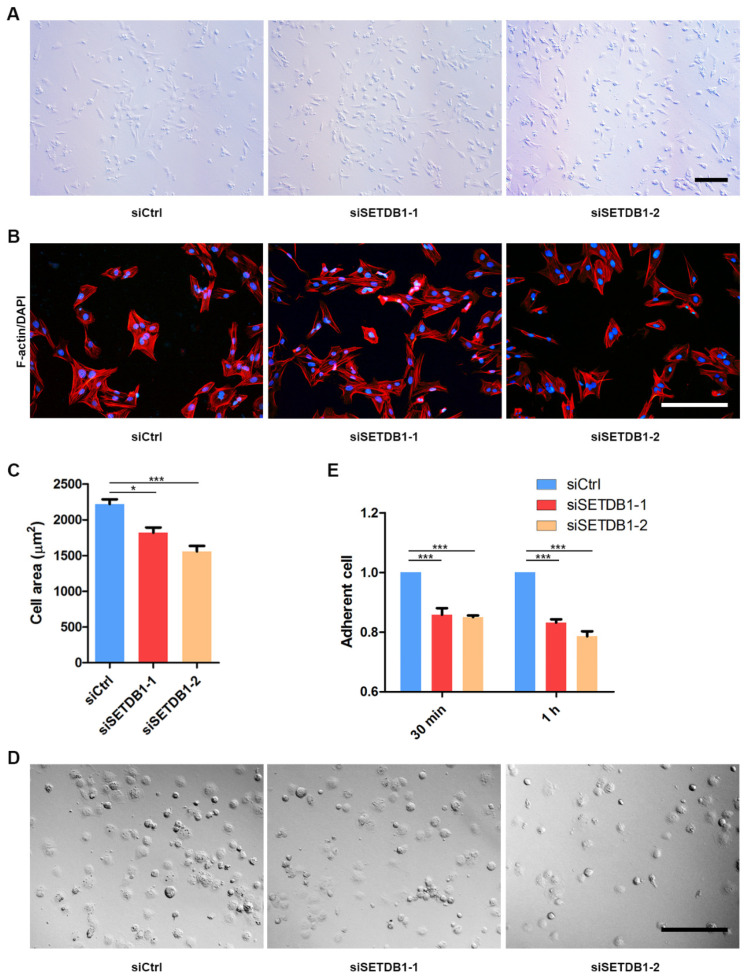
Knockdown of SETDB1 altered cell adhesion and cell spread. (**A**) Morphology of siSETDB1 and siCtrl cells. Scale bar = 100 μm. (**B**) Detection of cell spreading area by F-actin (red) and DAPI (blue) staining. Scale bar = 100 μm. (**C**) Analysis of cell area is shown in the column diagram. (**D**) Morphology of cells adhered for 30 min. Scale bar = 100 μm. (**E**) Detection of adherent cells in each group by CCK-8 assay. Significance was presented as * *p* < 0.05, *** *p* < 0.001. Error bars represented the standard error of the mean (SEM).

After homing to the niche, SSCs remain adhered to the basal membrane and to Sertoli cells. The developed spermatocytes then progressively migrate across the blood-testis barrier (BTB). We observed that many SETDB1-KD cells were fusiform or round, while siCtrl were mostly polygonal (Figure 4A). To probe whether cell spread was affected in SETDB1-KD cells, we stained actin filaments (F-actin) by phalloidin. The average spreading area of SETDB1-KD cells was significantly smaller than that in the siCtrl group (Figure 4B,C), indicating that deficiency in SETDB1 decreased cell spread. Consistently, the relative retaining rate of SETDB1-KD cells was significantly lower than that in the siCtrl group, as revealed by the cell-adhesion ability assay (Figure 4D,E).

**Figure 5:**
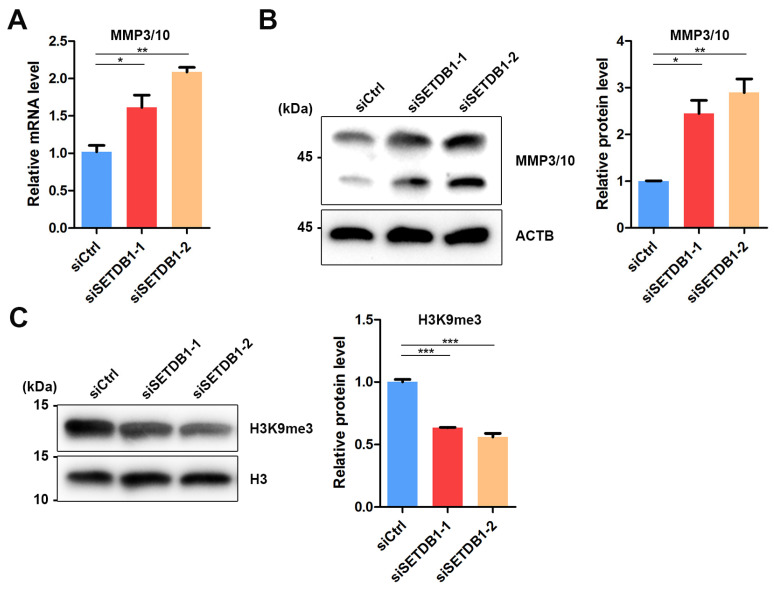
SETDB1 regulated MMP3/10 expression in porcine SSCs. (**A**) qRT-PCR analysis of the relative mRNA level of *MMP3/10* in porcine SSCs. (**B**) Western blot analysis of the protein levels of MMP3/10 and ACTB in porcine SSCs. The protein level of MMP3/10 relative to ACTB is shown in the column diagram. (**C**) Western blot analysis of the protein levels of H3K9me3 and histone H3 in porcine SSCs. The protein level of H3K9me3 relative to histone H3 is shown in the column diagram. Significance was presented as * *p* < 0.05, ** *p* < 0.01 or *** *p* < 0.001. Error bars represented the standard error of the mean (SEM).

Matrix metalloproteinases (MMPs) are responsible for the degradation of extracellular matrices (ECM), such as collagens, laminin and fibronectin, that constitute the niche for SSCs [43]. Previous studies have reported the abnormal expression of MMP10 in SETDB1-KO murine male PGCs [24]. Therefore, we speculated that SETDB1 regulates MMP3/10 expression in porcine SSCs. Indeed, a significant increase in MMP3/10 expression was observed in SETDB1-KO cells at both mRNA and protein levels (Figure 5A,B). Interestingly, the H3K9me3-dependent regulation of MMP3/10 is observed in mouse fibroblasts [44] and PGCs [24]. We found that the H3K9me3 level was significantly downregulated in SETDB1-KO cells (Figure 5C), consistent with the proposed function of SETDB1.

## 4. Discussion

SETDB1, a potential candidate to predict prognosis, has been well-studied in cancer cells. Although previous studies have revealed a variety of magnitudes in modulating spermatogenesis, research on SETDB and on its functions in SSCs has been limited by using mouse models. Due to the lack of efficient markers, it is difficult to isolate porcine SSCs. In addition, the low number of SSCs in testes and the suboptimal culture condition hinder the studies on SSCs from domestic animals. Here, by performing functional studies on an immortalized porcine SSC line, we identified that the loss of SETDB1 impaired cell proliferation and cell–cell adhesion in porcine SSCs.

The abnormal expression of SETDB1 predicts poor prognosis, as showed by various cancer research. Previous studies reported that SETDB1 was recruited to the promoter of Tumor Protein P53 (TP53) in colorectal cancer, promoting the proliferation and migration by inhibiting the TP53 expression [45]. High expression of SETDB1 was also identified in gastric cancer [38]. SETDB1 could also promote tumorigenesis and metastasis by interacting with ERG to upregulate cyclinD1 (CCND1) and MMP9 expression [38]. Anticancer drug treatment downregulated SETDB1 and increased FosB Proto-Oncogene (FOSB) expression, which was associated with cell proliferation during the therapy [46]. Interestingly, these studies showed the consistent oncogenic potential of SETDB1 overexpression and an opposite effect by suppression of SETDB1. In this study, the dysregulated proliferation and cell cycle process in SETDB1-KD SSCs were similar to the phenotypes in cancer cells. SETDB1 contains the Tudor domain to mediate protein–protein interactions [47], and the SET domain possesses the methyltransferase activity [48]. In addition to the histone lysine methyltransferase catalyzation, a previous study reported that SETDB1 also mediated the non-histone protein methylation [49]. Technically, SETDB1 deposited K64 methylation on the serine/threonine kinase Akt to facilitate the activation of Akt. SETDB1-mediated hyperactivation of Akt was identified to promote tumor development in non-small-cell lung carcinoma (NSCLC) [49]. These observations interpret why the SETDB1 expression is correlated with cell proliferation.

Homing is a basic property of SSCs. Gonocytes start transition to undifferentiated spermatogonia while migrating to the stem cell niche that is composed of basal membrane and Sertoli cells. Transplanted SSCs can migrate into the niche and reinitiate spermatogenesis for a long period of time [50]. We found that SETDB1 was highly expressed in gonocytes in postnatal porcine testes (7 d), in undifferentiated spermatogonia and spermatocytes at puberty (60 d), and in spermatocytes in adult testes (150 d). SETDB1 is dramatically upregulated during pachynema and is required for silencing of the XY Pair [25]. SYCP3 is the axial/lateral element of synaptonemal complex, which appears in the preleptotene spermatocytes situated at the basal membrane and maintains in pachytene spermatocytes situated at the inner layer of the seminiferous tubules [36]. Here, we found that the expression of SETDB1 in preleptotene spermatocytes was evident at puberty (60 d) but hardly detected at adult (150 d). We speculated that the exceptionally low proportion of SETDB1^+^/ SYCP3^+^ cells at 150 d could be ascribed to the high density of preleptotene spermatocytes.

ECM components and cell adhesion molecules have been identified to participate in the homing process and in maintenance of SSCs. An illustration of the ECM components is E-cadherin. E-cadherin, also known as CDH1, is specifically expressed in a subpopulation of spermatogonia including SSCs [51]. E-cadherin can regulate the interaction between germ cells and germ–Sertoli cells [52]. Another example of the ECM components is β1-integrin, which is produced by Sertoli cells and SSCs to mediate the homing process and cell location [11]. Interestingly, a previous report showed that SETDB1 deficiency in mouse PGCs upregulated genes related to ECM [24]. Of these ECM-related genes, *Mmp10* initiated transcription within an upstream long terminal repeat (LTR), which was suppressed by SETDB1 via H3K9me3 heterochromatin modification in control mice [24]. Consistently, we found that the expression of MMP3/10 in SETDB1-KD SSCs was upregulated at both mRNA and protein levels. Moreover, our unpublished ChIP-seq data on pubertal boar SSCs suggested that the pathways targeted by both SETDB1 and H3K9me3 were significantly enriched with cell adhesion. Notably, the expression of SETDB1 was gradually decreased in UCHL1^+^ SSCs and undifferentiated spermatogonia that are localized at the basal compartment of the tubules. The visible morphological changes and the disappeared SETDB1 staining in UCHL1^+^ cells were in line with the altered cell skeleton in SETDB1-KD SSCs.

Based on these data, we propose that SETDB1 regulates cell adhesion in SSCs by deposition of H3K9me3. SETDB1 suppresses MMP3/10 expression in SSCs prior to the emergence of multiple layers of germ cells. Then, the expression of SETDB1 in SSCs is progressively lost, due to the emergence of the blood-testis barrier that is formed by the tight junctions between Sertoli cells, limiting the migration of SSCs. Clearly, such noticeable gene expression and morphological difference between postnatal and adult undifferentiated spermatogonia is driven by many factors, and elucidating the underlying mechanisms would help to define the factors involved in niche–SSC interactions. Taken together, our results demonstrate that SETDB1 regulates the migration and proliferation of porcine SSCs, at least partially, through affecting the transcription of MMP3/10.

## Data Availability

The data presented in this study are available in the article or its Supplementary Material.

## Supplementary Materials

The following are available online at https://www.mdpi.com/article/10.3390/cells11030370/s1, Table S1: Oligo information.
