# Supplementary material for: SETDB1 Regulates Porcine Spermatogonial Adhesion and Proliferation through Modulating MMP3/10 Transcription
Source: Cells. 2022 Jan 22;11(3):370. doi: 10.3390/cells11030370 (PMC8834347; doi:10.3390/cells11030370)
Supplement: Supplementary file 1 [file cells-11-00370-s001.zip › cells-1518697-supplementary.pdf]

**Table S1. Oligo information.**

| Oligo name | Sequences (5′–3′) |
|---|---|
| siSETDB1-1-sense | GCUCCUUUGUCUGUAUUUATT |
| siSETDB1-1-anti | UAAAUACAGACAAAGGAGCTT |
| siSETDB1-2-sense | GGGAUUGCCAUUAAAUCAATT |
| siSETDB1-2-anti | UUGAUUUAAUGGCAAUCCCTT |
| siCtrl-sense | UUCUCCGAACGUGUCACGUTT |
| siCtrl-anti | ACGUGACACGUUCGGAGAATT |
| *HPRT1*-F | GAAGAGCTACTGTAATGACCAGTCAACGG |
| *HPRT1*-R | TCATTGTAGTCAAGGGCATAGCCTACC |
| *GAPDH*-F | ACACTCACTCTTCTACCTTTG |
| *GAPDH*-R | CAAATTCATTGTCGTACCAG |
| *SETDB1*-F | TGGATCTATCGAGGCTCTACACG |
| *SETDB1*-R | ACTGGACCACAGGACCTTTGC |
| *MMP3/10*-F | GGCCTGCCCAAGTGGAGAAA |
| *MMP3/10*-R | GCGGAGTCACTTCCTCCCAG |
